# Deregulated FASN Expression in BRAF Inhibitor-Resistant Melanoma Cells Unveils New Targets for Drug Combinations

**DOI:** 10.3390/cancers13092284

**Published:** 2021-05-10

**Authors:** Serena Stamatakos, Giovanni Luca Beretta, Elisabetta Vergani, Matteo Dugo, Cristina Corno, Elisabetta Corna, Stella Tinelli, Simona Frigerio, Emilio Ciusani, Monica Rodolfo, Paola Perego, Laura Gatti

**Affiliations:** 1 Department of Applied Research and Technological Development, Fondazione IRCCS Istituto Nazionale dei Tumori, 20133 Milan, Italy; serena.stamatakos@gmail.com (S.S.); Giovanni.Beretta@istitutotumori.mi.it (G.L.B.); Matteo.Dugo@istitutotumori.mi.it (M.D.); Cristina.Corno@istitutotumori.mi.it (C.C.); Elisabetta.Corna@istitutotumori.mi.it (E.C.); Stella.Tinelli@istitutotumori.mi.it (S.T.); Paola.Perego@istitutotumori.mi.it (P.P.); 2 Department of Research, Fondazione IRCCS Istituto Nazionale dei Tumori, 20133 Milan, Italy; Elisabetta.Vergani@istitutotumori.mi.it (E.V.); simona.frigerio@istitutotumori.mi.it (S.F.); 3 Department of Diagnostic and Technology, Fondazione IRCCS Istituto Neurologico Carlo Besta, 20133 Milan, Italy; emilio.ciusani@istituto-besta.it; 4 Department of Clinical Neurosciences, Fondazione IRCCS Istituto Neurologico Carlo Besta, 20133 Milan, Italy; laura.gatti@istituto-besta.it

**Keywords:** FASN inhibitor, vemurafenib, resistance, melanoma, DHCR24 inhibitor

## Abstract

**Simple Summary:**

Strategies to overcome resistance to targeted therapy represent a clinical need for metastatic melanoma. Altered lipid metabolism has been identified in the metabolic reprogramming associated with melanoma progression. Lipid metabolism genes such as fatty acid synthase (FASN) and 24-dehydrocholesterol reductase (DHCR24) have been proposed to contribute to tumor aggressiveness, but the therapeutic value of lipid metabolism inhibitors, particularly in drug-resistant melanoma, is unknown. Here, we found that molecular targeting of FASN in melanoma cells resistant to the BRAF inhibitor PLX4032 increased the sensitivity to the drug. Up-regulation of DHCR24 upon FASN targeting revealed the activation of druggable compensatory pathways sustaining the growth of resistant cells.

**Abstract:**

Metabolic changes promoting cell survival are involved in metastatic melanoma progression and in the development of drug resistance. In BRAF-inhibitor resistant melanoma cells, we explored the role of FASN, an enzyme involved in lipogenesis overexpressed in metastatic melanoma. Resistant melanoma cells displaying enhanced migratory and pro-invasive abilities increased sensitivity to the BRAF inhibitor PLX4032 upon the molecular targeting of FASN and upon treatment with the FASN inhibitor orlistat. This behavior was associated with a marked apoptosis and caspase 3/7 activation observed for the drug combination. The expression of FASN was found to be inversely associated with drug resistance in BRAF-mutant cell lines, both in a set of six resistant/sensitive matched lines and in the Cancer Cell Line Encyclopedia. A favorable drug interaction in resistant cells was also observed with U18666 A inhibiting DHCR24, which increased upon FASN targeting. The simultaneous combination of the two inhibitors showed a synergistic interaction with PLX4032 in resistant cells. In conclusion, FASN plays a role in BRAF-mutated melanoma progression, thereby creating novel therapeutic opportunities for the treatment of melanoma.

## 1. Introduction

Melanoma is the most aggressive skin tumor and a major cause of malignant skin cancer-related death [1]. A large number of patients develop distant metastases and less than 25% of them survive beyond 5 years from diagnosis [2]. Activating BRAF mutations present in 40–60% of melanoma patients induce aberrant activation of MAPK signaling, causing uncontrolled cellular growth [3]. The most frequent mutation found in around 90% of cases is V600 E. The discovery of vemurafenib (PLX4032) as a highly specific BRAFV600 E kinase inhibitor and its FDA-approval for treatment of BRAF mutant metastatic melanoma patients has changed the natural history of melanoma [4]. However, the efficacy of vemurafenib-based therapy is often limited by the occurrence of drug resistance [5]. MEK inhibitors have been introduced in a clinical setting, in combination with BRAF inhibitors (BRAFi) to obtain a more persistent control of the disease [6]. However, although BRAFi/MEKi-resistant patients can benefit from different therapeutic opportunities, such as immune checkpoint blockade, strategies aimed at impairing the onset of resistance by drug combinations remain an urgent unmet clinical need.

A large body of evidence supports that lipid metabolism is implicated in the development of cancer by promoting cell proliferation and survival and aggressive behavior [7], with changes in lipid metabolism favoring cell migration and invasion [8]. In tumor cells, the stimulation of lipid synthesis may result from the activation of oncogenic pathways, with newly synthesized lipids preferentially becoming phospholipids and participating in cell signaling [8]. Several melanoma driver genes, including mutated BRAF, have been shown to control cellular metabolism [9]. Cutaneous malignant melanoma and metastatic melanoma over-express fatty acid synthase (FASN), which converts dietary carbohydrates to long-chain saturated fatty acids from acetyl-CoA, malonyl-CoA and NADPH [10,11]. The overexpression of the lipogenic enzyme FASN has been associated with resistance to conventional antitumor agents [12]. Recent studies showed that altered lipid metabolism driven by oncogenic BRAF signaling contributed to targeted therapy resistance, which is mediated by SREBP1 activation [13] and by the upregulation of the S1 P-dependent signaling pathway [14]. These findings supported the use of SREBP1 or S1 P axis inhibitors in novel combination approaches to overcome resistance to BRAF inhibitors. Furthermore, manipulation of the expression of SREBP2 was shown to modulate the drug sensitivity of BRAFi refractory circulating metastatic melanoma cells by the regulation of iron homeostasis and ferroptosis [15].

Besides, among lipid metabolism regulatory genes, 24-dehydrocholesterol reductase (DHCR24, also known as seladin-1), a 3-hydroxysterol 24-reductase, catalyzes the terminal conversion of desmosterol in cholesterol. DHCR24 is required for cellular response to oncogenic and oxidative stress [16,17,18], is up-regulated in melanoma metastases compared to the corresponding primary tumor, and is associated with resistance to apoptosis [19]. Moreover, a link between DHCR24 and FASN in the context of the coordinated regulation of cholesterol biosynthesis and lipid metabolism genes has been described [20,21]. Thus, a better understanding of the role of such genes in the drug resistance of melanoma may be amenable for improving the efficacy of antitumor treatment [22].

Based on this background, the rationale of the study was designed to explore the possible role of FASN in relation to BRAFi resistance in melanoma cells, in order to improve the efficacy of BRAFi for combination studies. Inhibition of the lipid metabolism was achieved using both a molecular approach, i.e., transfection of small interfering RNAs (siRNA) and the FASN inhibitor orlistat, a well known anti-obesity drug [23], which has been shown to display antitumor effects [24] and has been already tested in clinical trials in cancer patients [25]. The effect of FASN targeting on DHCR24 expression revealed the activation of druggable compensatory pathways sustaining the growth of resistant cells.

## 2. Materials and Methods

### 2.1. Cell Lines and Cell Sensitivity to Drugs

Melanoma cell lines were derived from surgical specimens at the Fondazione IRCCS, Istituto Nazionale dei Tumori of Milan [26]. The PLX4032-resistant variants were generated in vitro upon exposure of parental cell lines to PLX4032, as previously described [27]. Cells were routinely authenticated using the Stem Elite ID System (Promega, Fitchburg, WI, USA), checked for mycoplasma contamination (Lonza, Basel, Switzerland) and used within 20 passages from thawing from frozen stock. The PLX4032-resistant cell lines were periodically tested to verify the maintenance of resistance to the selecting agent. All cell lines were cultured in RPMI-1640 medium (Lonza, Basel, Switzerland), supplemented with 10% FBS (Euroclone, Milan, Italy). The cell sensitivity to drugs was measured by a growth-inhibition assay based on cell counting (Z2 Particle Counter, Beckman Coulter, Milan, Italy), by the CCK8 (Sigma-Aldrich, St. Louis, MO, USA) or the lactate dehydrogenase (LDH) assays (Pierce, Waltham, MA, USA). For cell counting, exponentially growing cells were seeded into 12-well plates and, 24 h later, exposed to increasing concentrations of drugs for 72 h. For combination studies, the cells were treated according to 72 h concomitant exposure (simultaneous). For the CCK8 assay, cells were seeded in 96 well-plates (8000 cells/well) in 100 µL and 24 h, later exposed to drugs for 72 h. Cells were then added with the water-soluble formazan dye and cell viability was determined by reading optical density. For the LDH assay, which measures the release of LDH, cells were seeded in 96-well plates, and after 24 h, they were exposed to drugs for 72 h; cytotoxicity was determined according to the manufacturers’ protocol. All experiments were performed at least three times. IC_50_ is defined as the concentration of a drug inhibiting 50% of cell growth. The effect of drug combination was evaluated using the Chou–Talalay method, which allows one to assign a combination index (CI) value to each drug combination using the Calcusyn software (Biosoft, Cambridge, UK). CI values lower than 0.85–0.90 indicate synergistic drug interactions, whereas CI values higher than 1.20–1.45 or around 1 stand for antagonism or additive effect, respectively. PLX4032 (Selleckchem, Houston, TX, USA), orlistat and U18666 A (Cayman Chemical, Ann Arbor, MI, USA) were dissolved and diluted in DMSO. Final DMSO concentration in medium never exceeded 0.25%.

### 2.2. Loss of Function Studies

Cells were plated in 75 cm^2^ flasks (16,000 cells/cm^2^), and 24 h later, they were transfected using Opti-MEM transfection medium (Gibco by Thermo Fisher Scientific, Waltham, MA, USA) and Lipofectamine 3000 (Thermo Fisher Scientific, Waltham, MA, USA), with 10 nM of small interfering RNA (siRNA) to FASN (Silencer Select siRNA 5030 and 5032, Thermo Fisher Scientific, Waltham, MA, USA), in combination or singly, and control siRNA (Silencer Select Negative Control #2 siRNA, Thermo Fisher Scientific, Waltham, MA, USA). The transfection mix was added to complete medium for 24 h and then it was replaced with cell medium. Knockdown efficiency was evaluated by Western blotting as indicated, 48, 72 and 144 h after transfection start. Cells were harvested 48 h after transfection start and were re-seeded in 96-well plates at a density of 8000 cells/well in 100 µL of medium. Twenty-four hours later, cells were exposed to drugs by adding 100 µL of PLX4032-containing medium to determine cell growth inhibition by a CCK8 assay (Sigma-Aldrich, St. Louis, MO, USA), performed 72 h after treatment.

### 2.3. In Silico Analysis

Mutational data, RNA-Seq RPKM values, reverse phase protein array (RPPA) data and IC_50_ values for melanoma cell lines included in the Cancer Cell Line Encyclopedia (CCLE) [28] were downloaded from CCLE website (https://portals.broadinstitute.org/ccle/data, accessed on 1 April 2020). RPKM values were log2-transformed after adding a constant value of 1. Correlation analysis was performed on BRAFV600 E-mutant melanoma cell lines using the Spearman’s correlation coefficient.

### 2.4. Western Blot Analysis

Western blot analysis was carried out as previously described [29]. Lysates were fractionated by SDS-PAGE and proteins were blotted on nitrocellulose membranes. Blots were pre-blocked in PBS containing 5% (*w*/*v*) dried no fat milk and then incubated overnight at 4 °C with antibodies to anti-FASN (Sigma-Aldrich, St. Louis, MO, USA), anti-DHCR24 (Cell Signaling Technology, Danvers, MA, USA), p27 kip1 (BD Biosciences, Franklin Lakes, NJ, USA) and Bim (Abcam, Cambridge, UK). Anti-actin (Sigma-Aldrich, St. Louis, MO, USA) or anti-β-tubulin (Abcam, Cambridge, UK) antibodies were used as control for loading. Antibody binding to blots was detected by chemo-luminescence (GE Healthcare, Chicago, IL, USA). Secondary antibodies were obtained from GE Healthcare. At least three independent experiments were performed. Band intensity quantification was performed using the ImageJ 1.47 v software.

### 2.5. Apoptosis Analysis

Apoptosis was evaluated by Annexin V-binding assay (Immunostep, Salamanca, Spain) in cells treated for 48 h with PLX4032, alone or in combination with orlistat or U18666 A, according to a simultaneous schedule. At the end of treatment, floating and adherent cells were harvested, washed with cold PBS and re-suspended in binding buffer (10 mM HEPES-NaOH, pH 7.4, 2.5 mM CaCl2, and 140 mM NaCl, Immunostep). A fraction of 10^5^ cells was incubated in binding buffer at room temperature in the dark for 15 min with 5 μL of FITC-conjugated Annexin V and 10 μL of 2.5 μg/mL propidium iodide (Immunostep, Salamanca, Spain). Annexin V binding was detected by flow cytometry. At least 10^4^ events/samples were acquired and analyzed using a specific software (CellQuestPro, BD Biosciences, Franklin Lakes, NJ, USA). The activation of caspase 8 and 3/7 was determined using luminescent Caspase Glo 8 and 3/7 assays (Promega, Fitchburg, WI, USA). Cells were seeded (8 × 10^3^ cells/well) in 96-well plates and treated with drugs for 72 h. Caspase activation was detected according to manufacturer’s instructions.

### 2.6. RNA Extraction and qRT-PCR Analysis

RNA was extracted from melanoma cells with the miRNeasy Mini kit (Qiagen, Hilden, Germany) and retrotranscribed with the High-Capacity cDNA Archive kit (Thermo Fisher Scientific, Waltham, MA, USA). Real time qRT-PCR was carried out in triplicate and run on the QuantStudio 7 Flex instruments (Thermo Fisher Scientific, Waltham, MA, USA), and analysis was performed using QuantStudio 6 and 7 Flex software. The results are presented as relative quantification (RQ) and are calculated as RQ = (2^−ΔΔCt^) ± RQ min/max when using the control samples as calibrators. IDT assays for FASN (222990460), SREBF1 (222990448), SREBF2 (222990436) and TaqMan assays for DHCR24 (Hs00234140_m1) and β-actin (4326315 E) were used.

### 2.7. Statistical Analysis

Statistical analyses were performed using the GraphPad PrismTM software (La Jolla, CA, USA). Student’s *t* test (two-tailed) or one way ANOVA followed by Bonferroni correction were used for an analysis of cell growth, migration, invasion, apoptosis and qRT-PCR data, as indicated.

## 3. Results

### 3.1. Knockdown of FASN in BRAFi-Resistant Melanoma Cells

**Figure 1:**
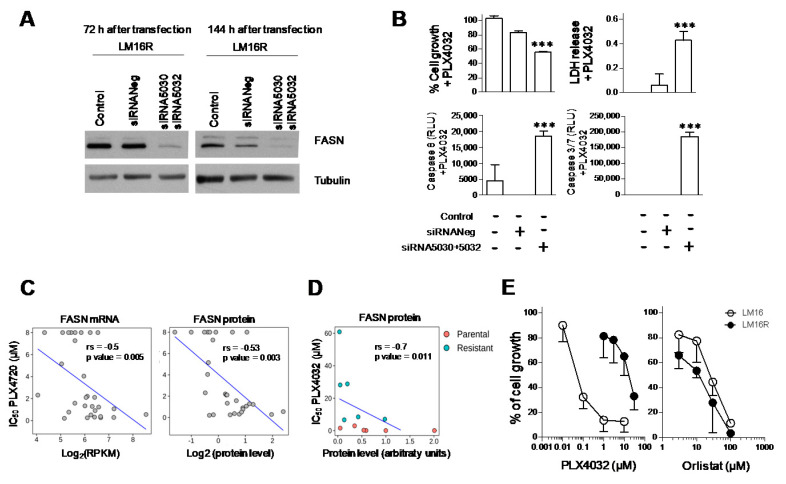
Expression of FASN in relation to resistance to BRAF inhibitors. (**A**) LM16 R cells were transfected with a mixture of two FASN-directed siRNAs (5 nM each, 10 nM final). The levels of FASN were evaluated by Western blotting 72 h after transfection, when drug treatment started, and at the end of drug treatment (144 h after transfection). Equal loading of SDS-PAGE is shown by tubulin. (**B**) Seventy-two hours after transfection, cells were analyzed for sensitivity to PLX4032 (3 µM) by CCK8 and LDH assays, and by caspase 3/7 and caspase 8 activation assays. *** *p* < 0.0001 by one-way ANOVA followed by Bonferroni correction. (**C**) Negative correlation between FASN mRNA (left) and protein (right) expression and IC_50_ values of PLX-4720 in BRAF-V600 E mutant melanoma cell lines from CCLE. (**D**) Negative correlation between FASN protein expression and IC_50_ values of PLX4032 in six sensitive (parental) and resistant cell lines. Spearman’s correlation coefficients and *p* values are indicated for each plot. (**E**) Sensitivity of melanoma cells to PLX4032 and to the FASN inhibitor orlistat. IC_50_ values of orlistat for LM16 R and LM16 parental cells were 12.40 µM ± 3.2 and 27.43 µM ± 5.3, *p* = 0.0138 by unpaired Student’s t test. IC_50_ values for PLX4032 were 11.62 ± 4.6 and 0.08 ± 0.04 µM.

FASN has been shown to be expressed in melanoma [10,11], but its relation to drug resistance is not known. Therefore, we examined the effect of the molecular inhibition of FASN in the BRAFi-resistant cells. A marked down-regulation of FASN protein levels was obtained upon transfection of two specific siRNAs (Figure 1A, Figures S1A and S5). Western blot assays performed 72 h after transfection, when cells were exposed to PLX4032, and at the end of the experiment, confirmed a marked decrease of the FASN protein. Transfected LM16 R cells were analyzed in terms of sensitivity to PLX4032 by CCK8 and LDH assays, as well as by caspase 8 and caspase 3/7 activation assays (Figure 1B and Figure S1B). The molecular inhibition of FASN resulted in increased cell growth inhibition and apoptosis activation after PLX4032 treatment.

### 3.2. Association between Dysregulation of FASN and Resistance to BRAF Inhibitors

Based on the above-reported finding, to assess whether the dysregulation of FASN is involved in the acquisition of resistance to BRAFi, we examined thirty BRAF-mutated melanoma cell lines from the CCLE database [28] for which gene expression and drug sensitivity data are available (Table S1). Spearman’s correlation between gene expression data of FASN and the IC_50_ values of PLX4720 (a structurally distinct analog of PLX4032) across BRAF-mutated melanomas indicated that FASN levels were inversely correlated with IC_50_ values both at gene (rs = −0.5) and protein level (rs = −0.53) (Figure 1C). Consistently, the inverse association between FASN protein levels and resistance to BRAFi treatment was confirmed in a panel of six matched resistant/sensitive cell lines treated with PLX4032 in cell growth inhibition assays (rs = −0.7) (Figure 1D), as FASN protein levels were reduced in the resistant variants when compared to their sensitive counterparts (Figure S2A). The negative correlation of FASN levels with BRAFi IC_50_ values supported the downregulation of FASN expression associated with resistance to BRAFi, pointing to a role for FASN regulation in the effectiveness of BRAFi therapy in melanoma.

### 3.3. Phenotype of LM16 R Cells Associates to Sensitivity to Orlistat

Since the highest levels of FASN were shown in the LM16 line compared to all the other cell lines and LM16 R cells demonstrated a significative FASN reduction, to assess the importance of FASN in sustaining cell growth, we examined their sensitivity to the FASN inhibitor orlistat by growth-inhibition assays following 72 h drug exposure. LM16 R cells, exhibiting high resistance to PLX4032 displayed collateral sensitivity to orlistat treatment, which showed a higher effect in LM16 R cells compared to LM16 cells (*p* = 0.0138 by unpaired Student’s *t* test) (Figure 1E).

### 3.4. Analysis of Cell Response to the Combination of PLX4032 and Orlistat

We sought to define whether the combination of orlistat and PLX4032 results in a favorable drug interaction. Using a simultaneous 72 h combination treatment with increasing concentrations of PLX4032 and a subtoxic concentration of the FASN inhibitor, we observed a synergistic interaction, both in LM16 R and LM16 cells (Table 1 and Table S2). The combination with the IC_50_ concentrations of PLX4032, respectively 10 µM and 0.1 µM, showed a synergistic effect for both cell lines. The best CI values were obtained in LM16 R cells exposed to 30 µM PLX4032 with 3 µM orlistat and in LM16 cells exposed to 0.1 µM PLX4032 with 3 µM orlistat.

**Figure 2:**
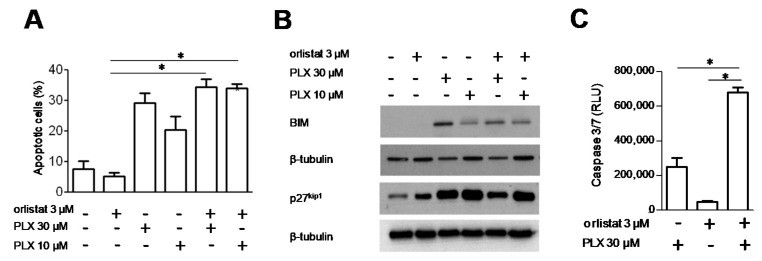
Effect of the combination of PLX4032 and orlistat in LM16 R cells. (**A**) Cells were exposed to single agents or to their combination and harvested 48 h after treatment for analysis of apoptotic response. Apoptosis was assessed by Annexin V-binding assay. Histograms represent the mean ± SEM of 3 independent experiments. * *p* < 0.05 by one-way ANOVA followed by Bonferroni correction. (**B**) Western blot analyses were carried out in exponentially growing cells. Cells were exposed to the drugs alone or in combination for 48 h and harvested for protein extraction. Samples were loaded on SDS-PAGE. Equal loading is shown by tubulin. (**C**) Caspase 3/7 activation evaluated in cells treated with PLX4032 alone or in combination with orlistat, by subtracting the untreated control. * *p* < 0.05 by one-way ANOVA followed by Bonferroni correction compared to single agents. The detected levels were significantly increased for all the treatments compared to untreated control (*p* < 0.05).

Cell response to the combination of PLX4032 and orlistat was examined in terms of apoptotic cell death, using flow-cytometric analysis by Annexin V-binding assays (Figure 2A and Figure S3A). In spite of the CI values observed, no significant increase of the fraction of Annexin-positive cells was achieved upon combined treatment in LM16 R and LM16 cells when compared to PLX4032 alone. Using western blot analyses, we evaluated the possible modulation of proteins involved in cell response. In LM16 cells, combination treatment resulted in no appreciable changes of Bax, Bcl-2, p21 ^waf1^ and p53 protein levels, while these proteins were undetectable in LM16 R cells (not shown). Moreover, no PARP1 cleavage was observed in either of the cell lines following drug exposure. In contrast, PLX4032 treatment produced an increased expression of BIM and p27 ^kip1^ in LM16 R and LM16, although no substantial modulations were evidenced for both the proteins when cells were exposed to the PLX4032/orlistat combinations (Figure 2B, Figures S3B and S6). When the activation of caspases was investigated using a caspase 3/7 luminescent assay, we observed that the combined treatment of PLX4032 with orlistat resulted in a significant activation of caspase 3/7 compared to single agent exposure only in LM16 R cells (Figure 2C and Figure S3C).

### 3.5. DHCR24 Modulation upon FASN Inhibition and the Effect of DHCR24 Targeting

**Figure 3:**
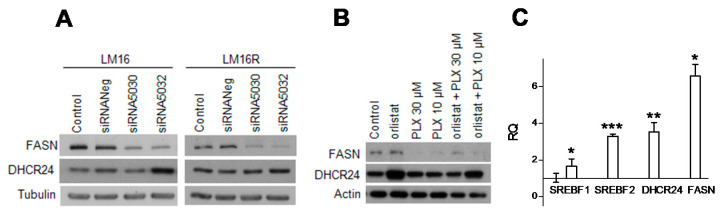
Effects of FASN inhibition on DHCR24 regulation. (**A**) DHCR24 levels evaluated by Western blot upon knockdown of FASN by RNA interference in LM16 at 48 h and in LM16 R cells at 72 h. Equal loading is shown by tubulin. (**B**) FASN and DHCR24 levels in LM16 R cells after 48 h treatment with orlistat (3 µM) and PLX4032, as single agents or in combination. Equal loading is shown by actin. (**C**) Expression levels of SREBF1, SREBF2, DHCR24 and FASN as determined by qRT-PCR in LM16 R cells upon treatment with orlistat (3 µM). Relative quantification (RQ) values are shown.* *p* < 0.05, ** *p* < 0.01, *** *p* < 0.0001 by unpaired Student’s *t* test compared to the vehicle that is (DMSO)-treated.

Because LM16 R cells are characterized by increased invasive abilities (Figure S2B and Text S1), and we previously reported up-regulation of DHCR24 in metastasis [19], we examined DHCR24 protein levels in our panel of 6 cell line pairs and we observed that the LM16 pair displayed the highest levels (Figure S2A). Moreover, based on recent findings showing that the FASN loss triggers the compensatory upregulation of DHCR24 by increasing SREBP2 activity in human hepatocarcinoma cell lines [21], we examined the impact of FASN molecular inhibition on DHCR24 levels. We observed an enhancement of DHCR24 expression upon FASN mRNA knockdown by the specific siRNA 5032, both in LM16 and LM16 R cells (Figure 3A and Figure S7A,B). The same effect on DHCR24 levels was obtained upon FASN inhibition by orlistat, alone or in combination with PLX4032 in the resistant variant (Figure 3B, Figures S4A and S8). Furthermore, treatment with orlistat alone or in combination with PLX4032 downregulated FASN in both the cell lines, suggesting a role for this drug in regulating lipid biosynthesis. Consistently, treatment increased the expression levels of SREBF1, SREBF2 and DHCR24 transcripts in both cell lines; a similar effect was detectable also on FASN mRNA expression, contrary to the reduction in protein levels (Figure 3C and Figure S4B).

**Figure 4:**
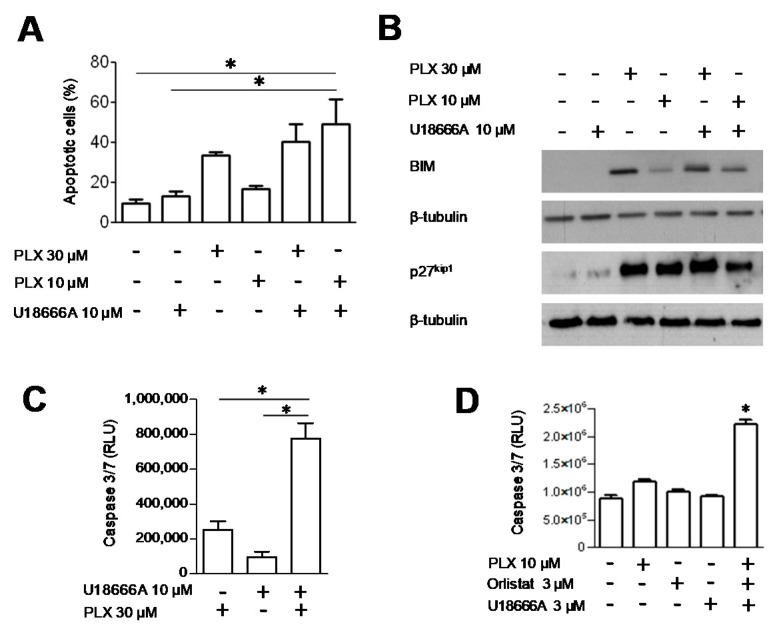
Effect of the combination of PLX4032 and U18666 A in LM16 R cells. (**A**) Apoptosis detection by Annexin V-binding assay upon treatment with U18666 A as single agent or combined with for 48 h. Histograms represent the mean ± SEM of 3 independent experiments. * *p* < 0.05 by one-way ANOVA followed by Bonferroni correction. (**B**) Western blot analyses of cells exposed to the drugs alone or in combination for 48 h and harvested for protein extraction. Equal loading is shown by tubulin. (**C**) Caspase 3/7 activation evaluated after treatment with PLX4032, alone or in combination with U18666 A, by subtracting the control level. * *p* < 0.05 by one-way ANOVA followed by Bonferroni correction compared to single agents. The levels were significantly increased for all the treatments compared to untreated control (*p* < 0.05). (**D**) Caspase 3/7 activation evaluated after treatment with PLX4032, orlistat and U18666 A, alone or in simultaneous combination for 72 h. * *p* < 0.05 by one-way ANOVA followed by Bonferroni correction compared to each single agent.

This cell behavior led us to test the effects of a chemical inhibitor of DHCR24, U18666 A [30], on BRAF sensitivity in melanoma cells. Using a simultaneous 72 h combination treatment with PLX4032 and U18666 A, we observed a synergistic interaction. The drug combination was particularly effective in LM16 R cells, the CI values being in the range 0.30–0.79 (Table 2). The combination increased the fraction of apoptotic cells (Figure 4A and Figure S3D). Exposure to U18666 A increased the levels of BIM and p27 ^kip1^, particularly in resistant cells (Figure 4B, Figures S3E and S9). In addition, the combined treatment resulted in a significant activation of caspase 3/7 in LM16 R cells compared to single agent exposure (Figure 4C and Figure S3F).

We then investigated the effects of a simultaneous 72 h combination treatment with PLX4032, orlistat and U18666 A. As reported in Table 3, a synergistic interaction was observed in LM16 R cells, with CI values ranging between 0.28 and 0.64. No relevant drug interaction was evidenced for LM16 cells (Table S3).

The activation of caspase 3/7 was evaluated in LM16 R cells after 72 h simultaneous exposure to 10 µM PLX4032, 3 µM orlistat and 3 µM U18666 A, a condition showing synergistic effects. We observed that the combined treatment resulted in a significant activation of caspase 3/7 compared to single agent exposure (Figure 4D). In contrast, no caspase 3/7 activation was detected for LM16 cells exposed to 0.1 µM PLX4032, 3 µM orlistat and 3 µM U18666 A.

## 4. Discussion

Resistance of melanoma cells to targeted agents including BRAFi is a multifactorial phenomenon that may involve lipid metabolism. Tumor cells are often characterized by increased energy-need and lipogenesis, with high fatty acid biosynthesis occurring independently of extracellular lipid levels [31]. FASN inhibition by orlistat has been reported to reduce proliferation and lymph node metastasis and to promote apoptosis in a mouse model of spontaneous melanoma metastasis [32]. In addition, an anti-apoptotic role has been shown for DHCR24 in neuronal cells [18], as well as in tumor cells [33]. Resistance to apoptosis may occur in BRAFi-resistant cells [27,34,35]. Thus, the interference with lipid metabolism by targeting lipogenesis may be exploited to increase the efficacy of BRAFi in melanoma, particularly in BRAFi resistant melanoma.

In this study, we first examined a possible effect of FASN knockdown on BRAFi efficacy in resistant cells and we found increased sensitivity, suggesting that FASN acts to protect cells from the growth inhibitory action of the drug, as an increased activation of caspase 8 and caspases 3/7 was found upon FASN knockdown. Then, we took advantage of the CCLE database to investigate the relationship between FASN levels and BRAFi resistance. In this setting, resistance to BRAFi in BRAF-mutant melanoma was associated with decreased FASN expression. The inverse relationship between FASN levels and resistance to BRAFi was also supported by protein analysis in six pairs of PLX4032-resistant and -sensitive cells. In fact, when FASN protein level was assessed in this panel of BRAFi-resistant cell lines and in their sensitive original counterparts by Western blotting, most cell lines expressed FASN, and most of the resistant variants exhibited FASN levels lower than their sensitive counterparts. LM16 cells displayed the highest FASN protein level and LM16 R showed reduction of the protein.

Based on the western blot analysis results, the LM16/LM16 R pair was considered suitable for evaluating the effects of PLX4032 in combination with orlistat. Of note, LM16 R displayed collateral sensitivity to orlistat, a behavior likely related to the reduced levels of FASN of the resistant variant with respect to the sensitive cell line. When the efficacy of the combination of orlistat and PLX4032 was assessed using subtoxic concentrations of orlistat, a synergistic drug interaction was observed at selected PLX4032 concentrations in both cell lines. The occurrence of a favorable interaction in resistant cells suggests that—although decreased in its level—FASN plays a key role also in survival of BRAFi-resistant cells. The combined treatment did not significantly increase apoptotic cells as compared to treatment with PLX4032 alone. The lack of an association between synergism and apoptosis induction by PLX4032 suggests that the interaction also occurs in terms of enhanced growth inhibition, an effect still potentially relevant from a therapeutic point of view to control the disease. This interpretation is in keeping with the results obtained from a biochemical analysis of cell response, indicating a marked up-regulation of the cyclin-dependent kinase inhibitor p27 ^kip1^ following exposure of cells to PLX4032. However, an increased expression of BIM was evidenced in sensitive and resistant cell lines exposed to the drug combinations, and this finding correlated with the increased activation of caspase 3/7 observed in resistant LM16 R cells. Thus, selected apoptosis-related proteins appeared to be induced by treatment. The promising features of targeting FASN are also supported by molecular targeting using siRNAs, as mentioned above. Molecular targeting of FASN resulted in the enhanced expression of DHCR24, although this effect was evident only with one siRNA, likely for kinetics reasons. Again, exposure of LM16 R cells to orlistat resulted in increased DHCR24 levels. However, the cell response to siRNAs that selectively inhibit genes is not fully superimposable to the effect obtained with drugs. It is possible that the acquisition of resistance to PLX4032 resulted in cellular changes, impacting cell response to both PLX4032 and orlistat. The two cell lines differ in the basal level of FASN and this feature may facilitate the achievement of any drug-induced down-regulation, including that triggered by PLX4032. It is true that there is no effect of orlistat on FASN levels in resistant cells, but in these cells, a marked up-regulation of DHCR24 was observed. Although the molecular details have not been clarified, these data suggest that BRAF inhibition in resistant cells might reduce survival signals coming from lipid metabolism-associated genes.

Compensatory changes upon the inhibition of single druggable genes should be taken into account, in accordance with recent findings showing that loss of FASN led to the increased cholesterol biosynthesis in a SREBP2-dependent manner in human hepatocarcinoma cells [21]. Similar findings were observed following the overexpression of miR-24 observed in hyperlipidemic conditions, determining decreased expression levels of FASN and ACLY genes and an increase of cholesterol synthesis genes including DHCR24, HMGCR and SREBP2 [20]. Consistently, we observed an up-regulation of SREBF1, SREBF2 and DHCR24 mRNA levels upon orlistat treatment, particularly in resistant cells. FASN mRNA levels were also increased, but a different effect was found in terms of protein expression in sensitive cells, likely due to the differential half life of mRNA/protein or to different exposure times. A DHCR24 increase may be still druggable, as shown in LM16 R cells exposed to the combination of the BRAFi with U18666 A and upon the simultaneous combination with U18666 A and orlistat, in which a synergistic interaction was found. Moreover, under these conditions, there was no significant enhancement in apoptotic cells when comparing single drug and combined treatment. Such an increase was observed in the parental cells that—however—displayed an additive interaction at best when using a PLX4032 concentration (1 µM) with a marked antiproliferative effect (around 80% of growth inhibition).

An additional observation made on resistant cells deserves attention. In fact, although DHCR24 has been associated with increased motility [19], LM16 R cells expressing it at lower levels compared to parental cells displayed increased invasive ability. It is likely that other factors than DHCR24 itself sustain the invasive behavior, as LM16 R cells were shown to display increased expression of metalloproteinases 2 and 9 and heightened Akt and ERK1/2 activation [36]. 

## 5. Conclusions

The dysregulation of lipid metabolism-related genes appears to be involved in the acquisition of resistance to BRAFi, with a decrease of FASN levels in resistant cells that can provide collateral sensitivity to FASN inhibition, a therapeutically relevant feature. Our findings show that the combination of PLX4032 with inhibitors of lipogenic enzymes results in favorable drug interactions potentially exploitable at the clinical level for the treatment of BRAFi-resistant melanoma. Of note, FASN inhibition has been reported to be selective for tumor cells that rely on the lipids synthesized by FASN for survival, with minimal adverse effects on normal cells. The gene expression and drug combination results reported here, together with the availability of well-tolerated FASN inhibitors, support the interest of clinical testing of FASN-targeting as a strategy to improve BRAFi efficacy in melanoma.

## Figures and Tables

**Table 1:** Analysis of the drug interaction between PLX4032 and orlistat in LM16 R cells ^1^.

Variable	CI ²	
PLX4032	3 µM orlistat	10 µM orlistat
30 µM	**0.22 ± 0.16**	**0.39 ± 0.11**
10 µM	**0.68 ± 0.07**	1.11 ± 0.35
3 µM	1.39 ± 0.3	1.40 ± 0.53
1 µM	4.3 ± 3.24	1.79 ± 0.62

^1^, Cell sensitivity was assessed by cell growth inhibition assay. Cells were seeded and 24 h later exposed to each drug and to their simultaneous combination for 72 h. Cells were then counted using a cell counter. ^2^, The drug interaction was analyzed by the Chou and Talalay method, calculating a combination index (CI). CI values indicating synergistic drug interactions are in bold. Mean CI values ± SE of three independent experiments are reported.

**Table 2:** Analysis of the drug interaction between PLX4032 and U18666 A in LM16 R cells ^1.^

Variable	CI ²	
PLX4032	3 µM U18666A	10 µM U18666A
30 µM	0.38 ± 0.15	0.30 ± 0.05
10 µM	0.44 ± 0.10	0.41 ± 0.04
3 µM	0.57 ± 0.18	0.67 ± 0.06
1 µM	0.55 ± 0.02	0.79 ± 0.04

^1^, Cell sensitivity was assessed by cell growth inhibition assay. ^2^ Combination Index (CI) values show synergistic drug interaction. CI are the mean ± SE of three independent experiments.

**Table 3:** Analysis of the drug interaction between PLX4032, orlistat and U18666 A in LM16 R cells ^1^.

PLX4032	Orlistat (µM)	U18666A (µM)	CI ^2^
10 µM	10 µM	10 µM	0.28 ± 0.17
10 µM	10 µM	3 µM	0.37 ± 0.36
10 µM	3 µM	10 µM	0.30 ± 0.26
10 µM	3 µM	3 µM	0.31 ± 0.05
3 µM	10 µM	10 µM	0.43 ± 0.00
3 µM	10 µM	3 µM	0.64 ± 0.12
3 µM	3 µM	10 µM	0.26 ± 0.25
3 µM	3 µM	3 µM	0.61 ± 0.26

^1^ Cell sensitivity was assessed by cell growth inhibition assay. ^2^ Combination Index (CI) values show synergistic drug interactions. CI are the mean ± SE of three independent experiments.

## Data Availability

The data that support the findings of our study are available from the corresponding author upon reasonable request.

## Supplementary Materials

The following are available online at https://www.mdpi.com/article/10.3390/cancers13092284/s1, Text S1: Cell Migration and Invasion Assay, Figure S1: Expression of FASN in relation to resistance to BRAF inhibitors, Figure S2: Expression of FASN and DHCR24 in relation to. resistance to BRAF inhibitors, Figure S3: Effect of the combination of PLX4032, orlistat and U18666A in LM16 cells, Figure S4: Effects of FASN inhibition on DHCR24 regulation in LM16 cells, Figure S5: Densitometric analysis was used to measure the band intensity (Figure 1A), Figure S6: Densitometric analysis was used to measure the band intensity (Figure 2B), Figure S7: Densitometric analysis was used to measure the band intensity (Figure 3A), Figure S8: Densitometric analysis was used to measure the band intensity (Figure 3B), Figure S9: Densitometric analysis was used to measure the band intensity (Figure 4B), Table S1: List of BRAF-mutated melanoma cell lines in CCLE database, Table S2: Analysis of the drug interaction between PLX4032 and orlistat or PLX4032 and U18666A in LM16 cells, Table S3: Analysis of the drug interaction between PLX4032, orlistat and U18666A in LM16 cells.
