# Supplementary material for: Deregulated FASN Expression in BRAF Inhibitor-Resistant Melanoma Cells Unveils New Targets for Drug Combinations
Source: Cancers (Basel). 2021 May 10;13(9):2284. doi: 10.3390/cancers13092284 (PMC8126202; doi:10.3390/cancers13092284)
Supplement: Supplementary file 1 [file cancers-13-02284-s001.zip › cancers-1186271-supplementary.pdf]

Supplementary Material: Deregulated FASN Expression in BRAF Inhibitor-Resistant Melanoma Cells Unveils New Targets for Drug Combinations

Serena Stamatakos, Giovanni Luca Beretta, Elisabetta Vergani, Matteo Dugo, Cristina Corno, Elisabetta Corna, Stella Tinelli, Simona Frigerio, Emilio Ciusani, Monica Rodolfo, Paola Perego and Laura Gatti

Text S1: Cell Migration and Invasion Assay

The assays were carried out as previously described, with minor modifications [1]. Cells were seeded (8 × 10^5 per well) to 24-well transwell chambers (Costar, Corning Inc., Corning, NY, USA) in serum-free medium; in the bottom well the complete medium was added. For invasion assays, the transwell membranes were previously coated with 12.5 µg matrigel per well (BD Biosciences, Franklin Lakes, NJ, USA) and dried for 1 h. After 24 h of incubation at 37 °C, cells that migrated to the lower chamber or invaded the matrigel and then migrated to the lower chamber were fixed in 95% ethanol, stained with a solution of 0.4% sulforhodamine B (SRB, Sigma–Aldrich, St. Louis, MO, USA) in 1% acetic acid, and counted under an inverted microscope.

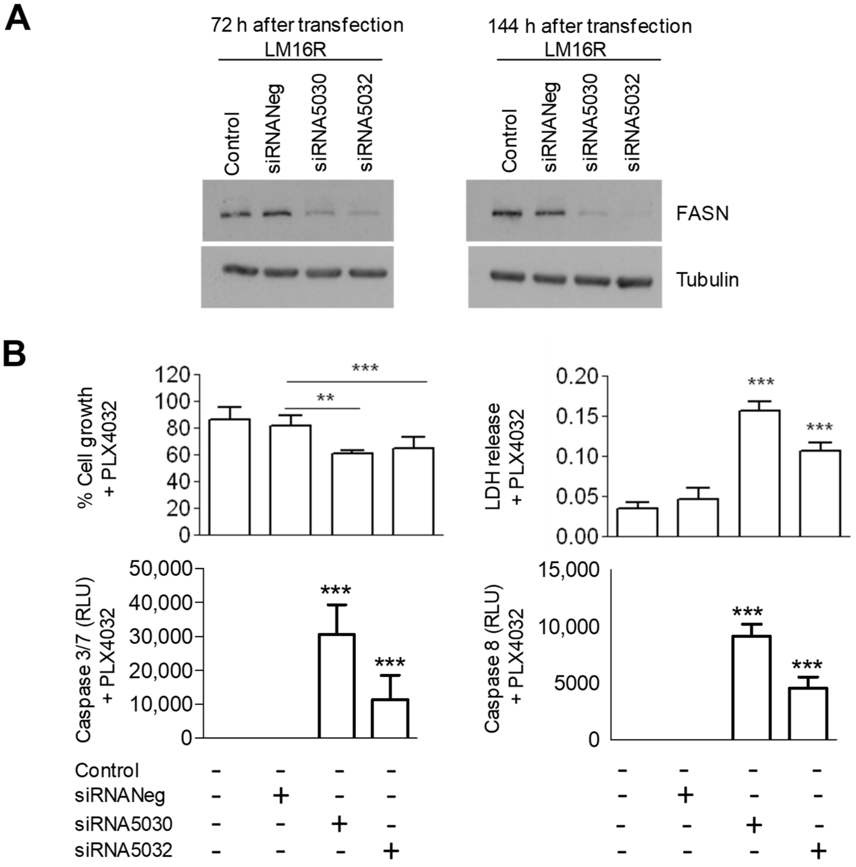

Figure S1. Expression of FASN in relation to resistance to BRAF. inhibitors. (**A**) Cells were transfected with FASN-directed siRNAs. The levels of FASN were evaluated by western blotting 72 h after transfection, when drug treatment started, and at the end of the experiment (144 h after transfection). Equal loading of SDS-PAGE is shown by tubulin. (**B**) Seventy two hours after transfection, cells were analyzed in terms of sensitivity to PLX4032 (3 μM) by CCK8 assay and LDH assay, and by caspase 3/7 and caspase 8 activation assays. ** $P < 0.01$, *** $P < 0.0001$ by one-way ANOVA followed by Bonferroni correction.

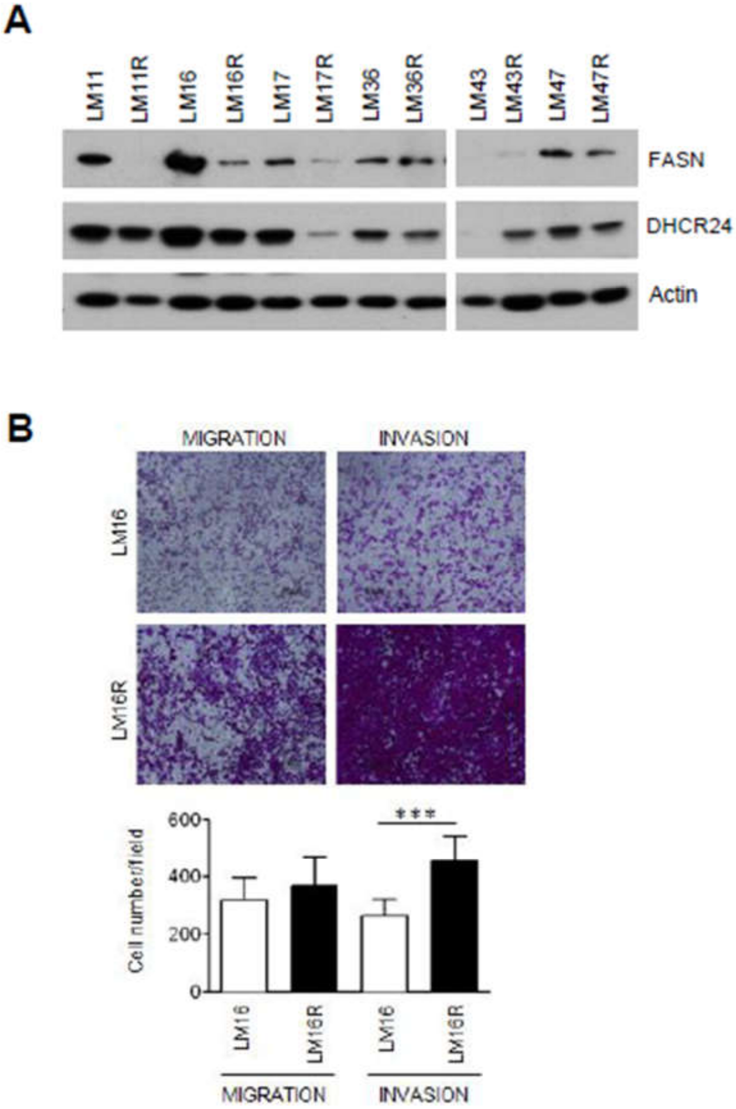

Figure S2. (**A**) Expression of FASN and DHCR24 in relation to. resistance to BRAF inhibitors. Western blot analysis of FASN and DHCR24 levels in sensitive and resistant cell lines. Equal loading is shown by actin. (**B**) Representative images of migration and invasion assays in serum-free medium using transwell plates. Migrating and invading cells were counted under a light microscope. Columns represent cell numbers/field (± SE; $n = 3$). *** $P < 0.001$ by unpaired Student's t test.

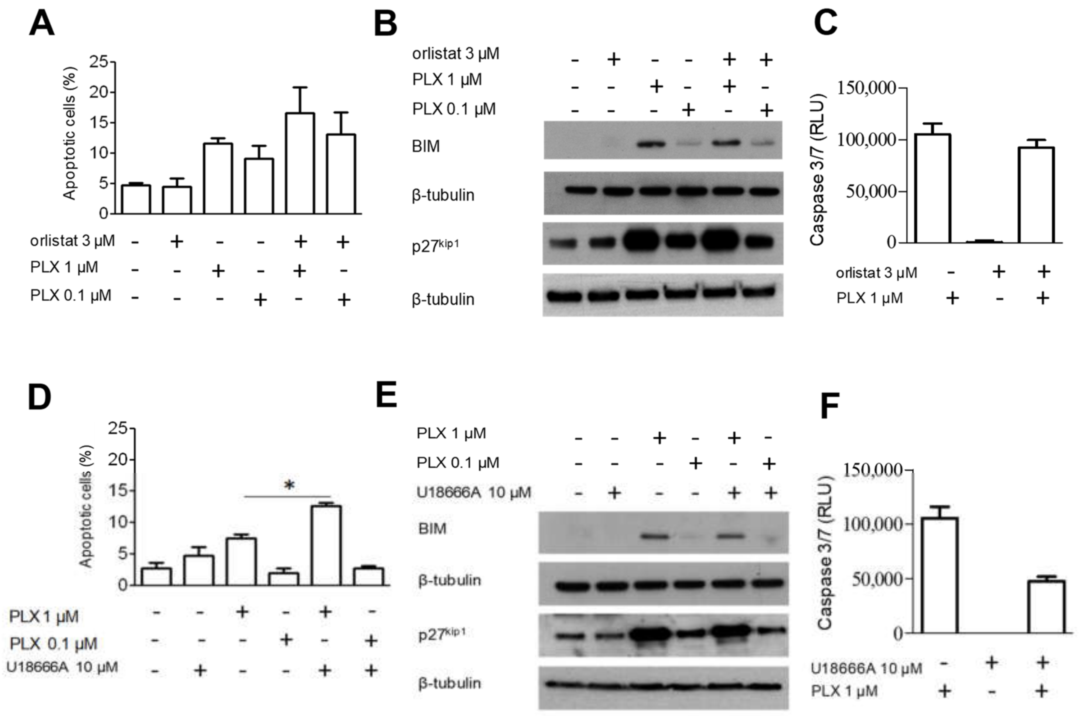

Figure S3. Effect of the combination of PLX4032, orlistat and U18666A in LM16 cells. (**A**–**D**), LM16 cells were exposed to single agents or to their combination and harvested 48 h after treatment for analysis of apoptotic response by Annexin V-binding assay. * $P < 0.05$ by one-way ANOVA followed by Bonferroni correction. (**B**–**E**), Western blot analyses in LM16 cells exposed to the drugs alone or in combination for 48 h. Equal loading is shown by tubulin. (**C**–**F**), Caspase 3/7 activation evaluated in LM16 cells treated with PLX4032 alone or in combination with orlistat or U18666A by subtracting the control levels. * $P < 0.05$ by one-way ANOVA followed by Bonferroni correction compared to single agents.

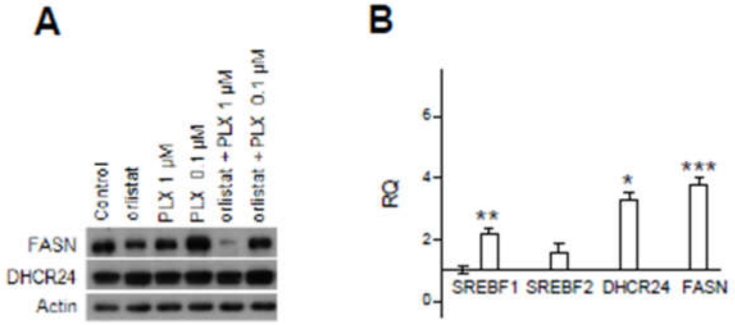

Figure S4. Effects of FASN inhibition on DHCR24 regulation in LM16 cells. (**A**) FASN and DHCR24 levels after 48 h treatment with orlistat (3 µM) and PLX4032 as single agents or in combination. Equal loading is shown by actin. (**B**) Expression levels of SREBF1, SREBF2. DHCR24 and FASN as determined by qRT-PCR in LM16 cells upon treatment with orlistat (3 µM). Relative quantification (RQ) values are shown.* $P < 0.05$, ** $P < 0.01$, *** $P < 0.0001$ by unpaired Student's t test compared to the vehicle (DMSO)-treated.

Table S1. List of BRAF-mutated melanoma cell lines in CCLE database.

CCLE_ID	depMapID	Name	Histology	BRAF_mutation	PLX4720 IC50 (µM)
A2058_SKIN	ACH-000788	A2058	malignant_melanoma	p.V600E	8
A375_SKIN	ACH-000219	A-375	malignant_melanoma	p.V600E	0.258287698
C32_SKIN	ACH-000580	C32	malignant_melanoma	p.V600E	2.206977606
COLO679_SKIN	ACH-000805	COLO-679	malignant_melanoma	p.V600E	0.554165304

COLO741_SKIN	ACH-000582	COLO 741	malignant_melanoma	p.V600E	4.03970623
G361_SKIN	ACH-000572	G-361	malignant_melanoma	p.V600E	1.286707997
HS294T_SKIN	ACH-000014	Hs 294T	malignant_melanoma	p.V600E	8
HS695T_SKIN	ACH-000799	Hs 695T	malignant_melanoma	p.V600E	7.264561176
HS939T_SKIN	ACH-000814	Hs 939.T	malignant_melanoma	p.V600E	2.102822304
HT144_SKIN	ACH-000322	HT-144	malignant_melanoma	p.V600E	1.336324811
IGR37_SKIN	ACH-000650	IGR-37	malignant_melanoma	p.V600E	0.904429674
IGR39_SKIN	ACH-000550	IGR-39	malignant_melanoma	p.V600E	8
K029AX_SKIN	ACH-000404	K029AX	malignant_melanoma	p.V600E	2.110589266
LOXIMVI_SKIN	ACH-000750	LOX IMVI	malignant_melanoma	p.V600E;p.I208V	8
MALME3M_SKIN	ACH-000477	Malme-3M	malignant_melanoma	p.V600E	0.285528094
MDAMB435S_SKIN	ACH-000884	MDA-MB-435S	malignant_melanoma	p.V600E	2.312842846
MELHO_SKIN	ACH-000450	MEL-HO	malignant_melanoma	p.V600E	0.312976211
RPMI7951_SKIN	ACH-000348	RPMI-7951	malignant_melanoma	p.V600E	8
RVH421_SKIN	ACH-000614	RVH-421	malignant_melanoma	p.V600E	0.770638406
SKMEL24_SKIN	ACH-000822	SK-MEL-24	malignant_melanoma	p.V600E	5.15450716
SKMEL31_SKIN	ACH-000640	SK-MEL-31	malignant_melanoma	p.V600E	8
SKMEL5_SKIN	ACH-000730	SK-MEL-5	malignant_melanoma	p.V600E	0.369111031
UACC257_SKIN	ACH-000579	UACC-257	malignant_melanoma	p.V600E	1.064142346
UACC62_SKIN	ACH-000425	UACC-62	malignant_melanoma	p.V600E	0.249700621
WM115_SKIN	ACH-000304	WM-115	malignant_melanoma	p.V600V;p.V600E;p.V600D	8
WM1799_SKIN	ACH-000661	WM1799	malignant_melanoma	p.V600E	1.226397991
WM2664_SKIN	ACH-001239	WM-266-4	malignant_melanoma	p.V600V;p.V600E;p.V600D	1.578695059
WM793_SKIN	ACH-000827	WM-793	malignant_melanoma	p.V600E	8
WM88_SKIN	ACH-000899	WM-88	malignant_melanoma	p.V600E	0.204083994
WM983B_SKIN	ACH-000765	WM-983B	malignant_melanoma	p.V600E	0.51123476

Table S2. Analysis of the drug interaction between PLX4032 and orlistat or PLX4032 and U18666A in LM16 cells[1].

-	CI[2]			
**PLX4032**	**3 μM orlistat**	**10 μM orlistat**	**3 μM U18666A**	**10 μM U18666A**
10 μM	2.97 ± 0.81	4.53 ± 3.00	2.67 ± 1.23	3.40 ± 0.78
1 μM	**0.6 ± 0.18**	**0.67 ± 0.40**	**0.72 ± 0.10**	1.17 ± 0.19
0.1 μM	**0.23 ± 0.18**	0.94 ± 0.68	1.68 ± 1.14	2.14 ± 0.68
0.01 μM	31.06 ± 12.75	3.34 ± 0.52	17.35 ± 19.85	2.83 ± 0.88

[1], Cell sensitivity was assessed by cell growth inhibition assay. Cells were seeded and 24 h later exposed to each drug and to their simultaneous combination for 72 h. Cells were then counted using a cell counter. [2], The drug interaction was analyzed by the Chou and Talalay method, calculating a combination index (CI) and CI values indicating synergistic drug interactions are in bold. Mean CI values ± SE of three independent experiments are reported.

Table S3. Analysis of the drug interaction between PLX4032, orlistat and U18666A in LM16 cells[1].

PLX4032	orlistat (μM)	U18666A (μM)	CI[2]
0.1 μM	10 μM	10 μM	1.11 ± 0.13
0.1 μM	10 μM	3 μM	**0.67 ± 0.07**
0.1 μM	3 μM	10 μM	1.4 ± 0.96
0.1 μM	3 μM	3 μM	**0.41 ± 0.02**
0.01 μM	10 μM	10 μM	0.96 ± 0.18
0.01 μM	10 μM	3 μM	1.06 ± 0.32
0.01 μM	3 μM	10 μM	0.86 ± 0.21
0.01 μM	3 μM	3 μM	4.90 ± 3.60

[1], Cell sensitivity was assessed by cell growth inhibition assay. [2], Combination Index (CI) values showing synergistic drug interactions are reported in bold. CI are the mean ± SE of three independent experiments.

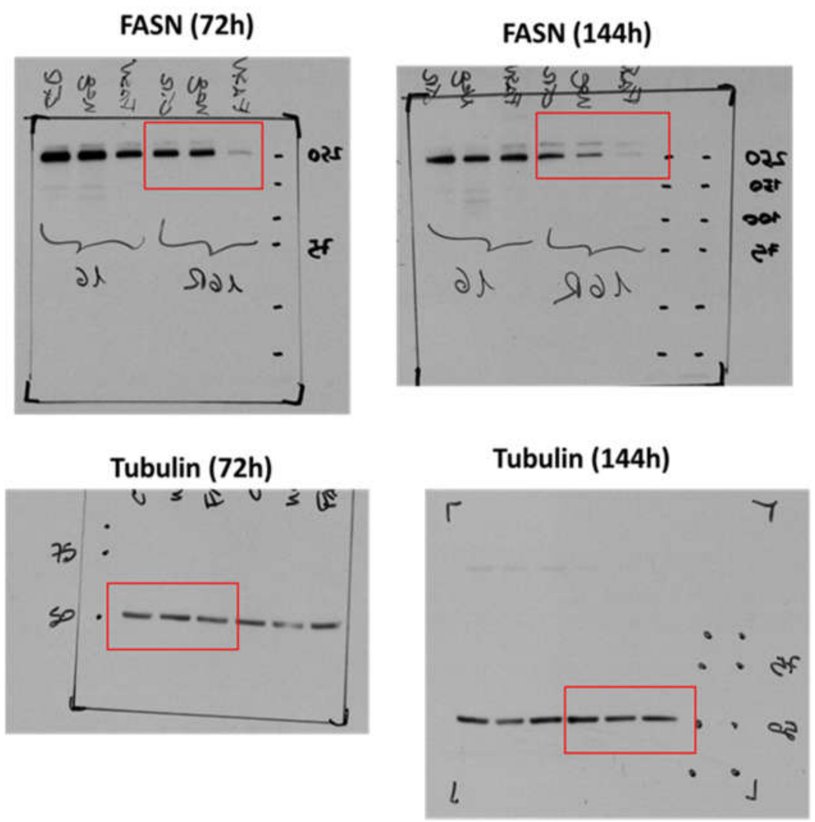

Name	FASN	Tubulin	FASN/Tub
LM16R CTR 72h	127426020	74338867	1.714123784
LM16R siRNANeg 72h	124549035	71666862	1.737888775
LM16R siFASN 72h	35576396	63207505	0.56285082
LM16R CTR 144h	142853257	101872687	1.402272397
LM16R siRNANeg 144h	106147120	71882698	1.47667134
LM16R siFASN 144h	10724711	71544124	0.14990345

Figure S5. Densitometric analysis was used to measure the band intensity (Figure 1A).The relative expression levels (ratio between the band intensity of FASNand the corresponding band intensity of tubulin) was determined by ImageQuant 5.2 software.

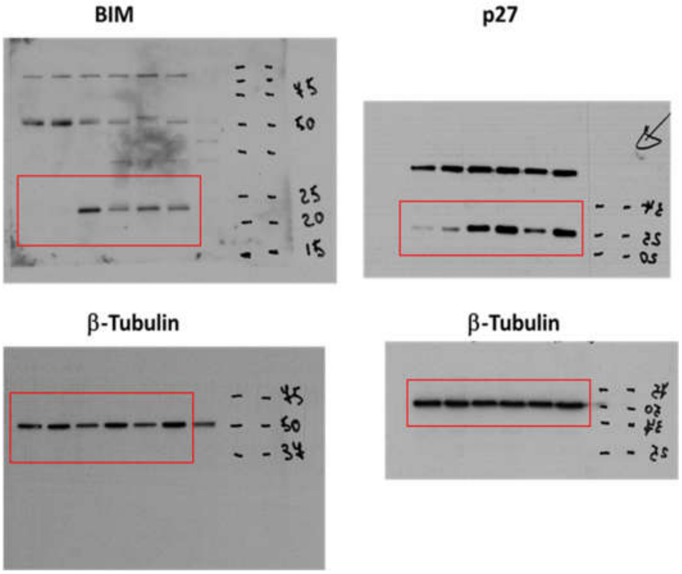

Name	BIM	Tubulin	BIM/Tub	p27	Tubulin	p27/Tub
Control	2010433	32166158	0.062501	30510668	136545134	0.223447
Orlistat 3µM	2072734	43911430	0.047203	49963595	127961429	0.390458
PLX 1µM	29204735	30437774	0.95949	120242560	127325274	0.944373
PLX 0,1µM	26407246	35806485	0.737499	140637886	134499052	1.045642
Orlistat 3µM/PLX 1µM	30158109	27188883	1.109207	74996051	126729961	0.591778
Orlistat 3µM/PLX 0,1µM	23825512	39033819	0.610381	127618264	146163452	0.87312

Figure S6. Densitometric analysis was used to measure the band intensity (Figure 2B).The relative expression levels (ratio between the band intensity of BIM or p27and the corresponding band intensity of tubulin) was determined by ImageQuant 5.2 software.

A

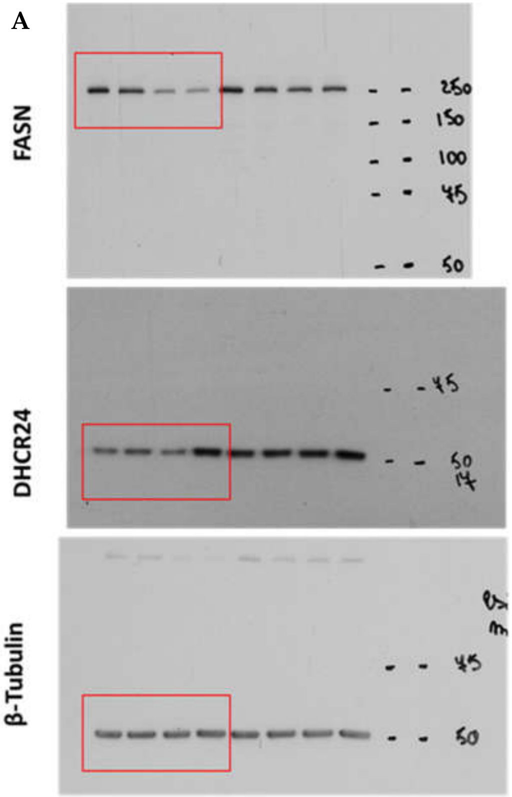

Name	FASN	Tubulin	DHCR24	FASN/Tub	DHCR24/Tub
Control	23558677	21766672	15567568	1.082327928	0.71520203
siRNANeg	25182525	22490675	15473418	1.119687382	0.687992601
siRNA5030	18079820	21400241	13068710	0.844841888	0.610680506
siRNA5032	20323428	29542737	29002517	0.687933146	0.981713949

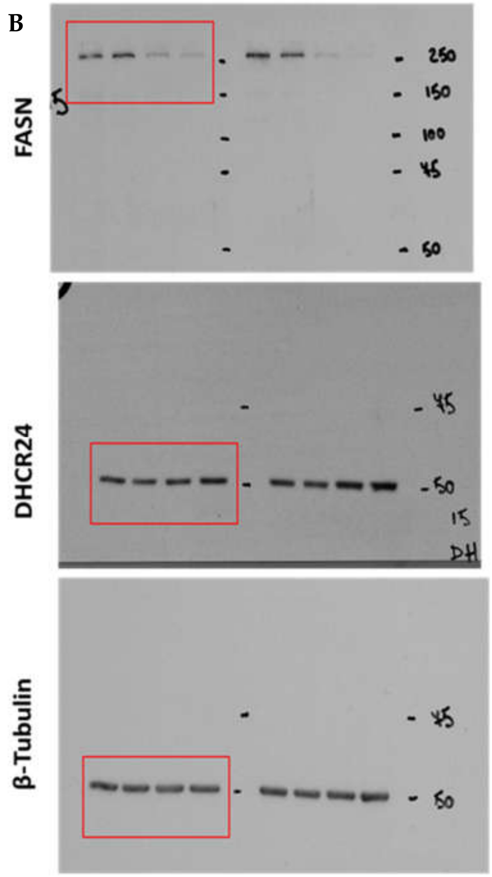

Name	FASN	Tubulin	DHCR24	FASN/Tub	DHCR24/Tub
Control	25743965	20275024	35150003	0,732402925	0,576814289
siRNANeg	28574255	20326075	37276740	0,766543829	0,545275016
siRNA5030	11068424	20005782	33110329	0,334289158	0,604215742
siRNA5032	10372737	28552974	34187096	0,303410883	0,835197409

Figure S7. Densitometric analysis was used to measure the band intensity (Figure 3A). (**A**–**B**)The relative expression levels (ratio between the band intensity of FASN or DHCR24 and the corresponding band intensity of tubulin) was determined by ImageQuant 5.2 software.

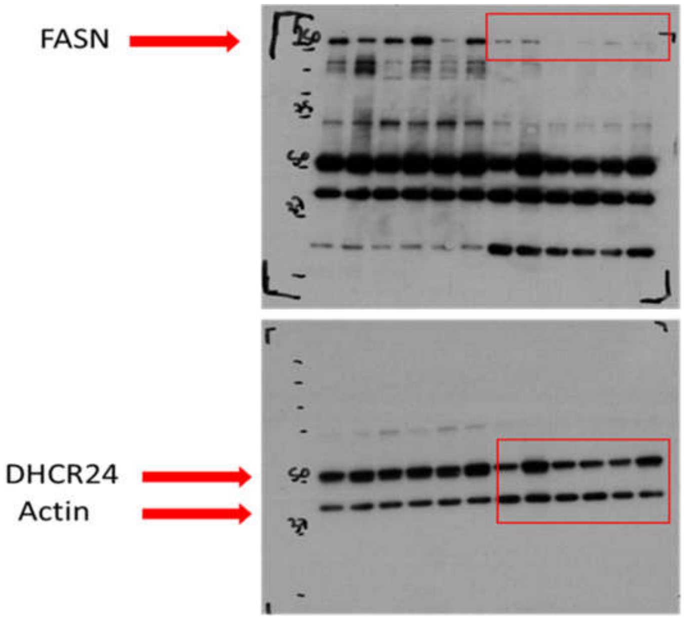

Name	FASN	Actin	DHCR24	FASN/Act	DHCR24/Act
**Control**	**1723548**	**16907673**	**16469137**	**0.101939**	**0.974063**
**Orlistat 3μM**	**2438740**	**17332794**	**27145421**	**0.140701**	**1.566131**
**PLX 30μM**	**72778**	**16779087**	**17629673**	**0.004337**	**1.050693**
**PLX 10μM**	**280213**	**17230945**	**14398530**	**0.016262**	**0.83562**
**Orlistat 3μM/PLX 30μM**	**753527**	**13037288**	**12562238**	**0.057798**	**0.963562**
**Orlistat 3μM/PLX 10μM**	**151607**	**11949388**	**21196815**	**0.012687**	**1.773883**

Figure S8. Densitometric analysis was used to measure the band intensity (Figure 3B). The relative expression levels (ratio between the band intensity of FASN or DHCR24 and the corresponding band intensity of actin) was determined by ImageQuant 5.2 software.

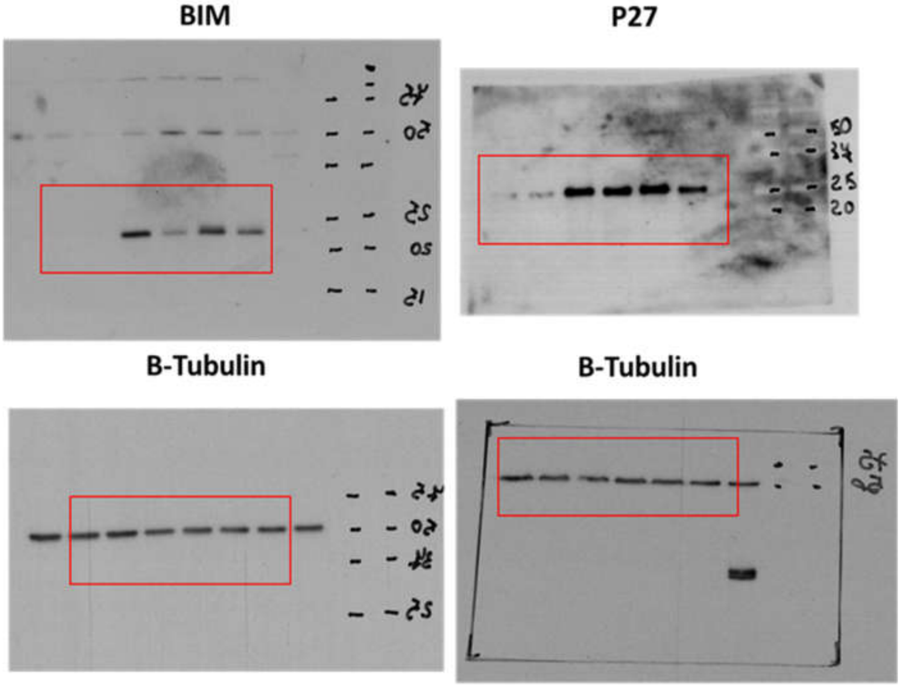

**Control**	**BIM**	**Tubulin**	**BIM/Tub**	**p27**	**Tubulin**	**p27/Tub**
	2042826	35578529	0.057417382	14543052	93805921	0.155033412
UO18666A 10μM	1865769	30854372	0.060470166	19177503	80930032	0.236963986
PLX 1μM	28865804	31682243	0.911103548	100724853	70466052	1.429409626
PLX 0,1μM	21921386	29733415	0.73726432	102535892	78837933	1.300590821
UO18666A 10μM/PLX 1μM	29006107	29194467	0.993548093	123746740	68595515	1.804006282
UO18666A 10μM/PLX 0,1μM	24540774	31639384	0.775640069	79370888	63025720	1.259341234

Figure S9. Densitometric analysis was used to measure the band intensity (Figure 4B). The relative expression levels (ratio between the band intensity of BIM or p27 and the corresponding band intensity of tubulin) was determined by ImageQuant 5.2 software.

References

1. Cassinelli, G.; Lanzi, C.; Petrangolini, G.; Tortoreto, M.; Pratesi, G.; Cuccuru, G.; Laccabue, D.; Supino, R.; Belluco, S.; Favini, E.; Poletti, A.; Zunino, F. Inhibition of c-Met and prevention of spontaneous metastatic spreading by the 2-indolinone RPI-1. *Mol Cancer Ther.* **2006**, *5*, 2388–2397, doi:10.1158/1535-7163.MCT-06-0245.

Publisher's Note: MDPI stays neutral with regard to jurisdictional claims in published maps and institutional affiliations.

© 2021 by the authors. Licensee MDPI, Basel, Switzerland. This article is an open access article distributed under the terms and conditions of the Creative Commons Attribution (CC BY) license (http://creativecommons.org/licenses/by/4.0/).
